# Synthesis and Molecular Descriptor Characterization of Novel 4-Hydroxy-chromene-2-one Derivatives as Antimicrobial Agents

**DOI:** 10.3390/molecules14041495

**Published:** 2009-04-14

**Authors:** Milan Mladenović, Nenad Vuković, Neda Nićiforović, Slobodan Sukdolak, Slavica Solujić

**Affiliations:** Department of Chemistry, Faculty of Science, University of Kragujevac, P.O. Box 60, Serbia; E-mails: nvukovic@kg.ac.rs (N.V.), nneda@kg.ac.rs (N.N.), duda@kg.ac.rs (S.S.), ssolujic@kg.ac.rs (S.S.)

**Keywords:** Coumarins, Microwave synthesis, Molecular descriptors, Antimicrobial activity

## Abstract

Several novel 4-hydroxy-chromene-2-one derivatives **2b**-**16b** were easily prepared through condensation reactions with microwave heating and characterized by elemental analysis, IR, ^1^H-NMR and mass spectrometry. Geometry optimization of these compounds was executed by PM3, PM5 and Minimize Energy methods to describe them via molecular descriptors. The antimicrobial activity of the synthesized compounds was evaluated against different microbial strains using two different methods: the diffusion method and the micro-dilution method. All data indicated that the products possess antimicrobial activity which depends on the nature of substituent attached to the benzopyran moiety. In general, after 24 h the MIC values of most tested coumarins was 0.13 mg/mL, but compounds **1** and **6b** displayed the strongest antimicrobial activity on the tested cultures of bacteria after 48 h. Compound **13b** has the strongest growth inhibitory potential on fungus *C. albicans*, tested by diffusion method, with an inhibition zone of 30-37 mm at a concentration of 150 µg/mL. The conclusion of this experiment is that the synthesized compounds have varied and different influence on different classes of bacteria and the fungus *C. albicans.*

## 1. Introduction

Coumarins are benzopyran derivatives. Naturally occurring coumarins have been isolated from over 800 species of plants and microorganisms [1], and many of these natural products exhibit useful drug-like activity [2,3]. Moreover, coumarins are a group of compounds that play important roles as food constituents, antioxidants, stabilizers and immunomodulatory substances, as fluorescent markers for use in analyses, in stains, and in clinical use [4,5]. Coumarins possess anti-inflammatory, antiallergic, hepatoprotective, spazmolitic, antiviral, anticarcinogenic and anticoagulant activities [6]. They also constitute an important group of organic compounds that are used as additives to cosmetics, as optical brightening agents, and dispersed fluorescent and laser dyes [7,8,9]. Coumarins also have important effects in plant biochemistry and physiology, acting as enzyme inhibitors and precursors of toxic substances. In addition, these compounds are involved in the actions of plant growth hormones and growth regulators, the control of respiration, photosynthesis, as well as defense against infection [10]. These natural aromatic compounds exhibit enormous structural variability, due to the various types of substitutions in their basic structure possible, which in turn can influence their biological activity. 

The first reports of the application of commercial microwave ovens to the synthesis of small organic molecules appeared in 1986. [11,12]. Microwave irradiation has since been proven to be extremely useful for promoting and simplifying many condensation reactions which can be carried out both in solvents and under solvent-free conditions [13,14,15,16,17]. Microwave technology is particularly suitable for the rapid and automated production of libraries of compounds, as it enables organic chemists to reduce the time of synthesis from days and hours to minutes and even seconds [18,19,20]. In addition, suppressed formation of side-products and improved yields under microwave heating conditions has frequently been observed. Finally, the proper choice of microwave processing techniques (solvent-free, solid- or polymer-supported conditions) can simplify the workup and avoid laborious and time-consuming purification of target compounds.

The essence of this work was synthesis of coumarin derivatives using microwave irradiation, in comparison with conventional methods. Coumarin derivatives have previously been studied in our laboratory [21,22,23,24,25,26]. We have now extended these studies to investigate their structural modification through Knoevenagel condensations carried out under microwave irradiation and leading to several 4-hydroxychromene-2-one derivatives. We also report a conventional and convenient microwave-promoted solution synthesis of eight imino derivatives of 4-hydroxychromene-2-one. All the reactions performed were monitored by TLC and GC/MS and yields were determined using the latter technique.

The use of solvent-free conditions for the fast synthesis of novel coumarin derivatives by the Knoevenagel condensation under microwave irradiation offers several advantages [27]: solvents are often expensive, toxic environmental polluting agents, and in the case of aprotic dipolar solvents with high boiling points, difficult to remove. Moreover, liquid-liquid extraction for the isolation of reaction products can be avoided, and the absence of solvent also avoids the risk of hazardous explosions when the reactions take place in a microwave oven. The workup procedure is reduced to the simple recrystallization of products from an appropriate solvent. The classical organic synthesis of imine derivatives commonly faces the problems of low yields, reaction byproducts, removal of solvents from reaction mixtures or liquid extraction, especially in the case of aprotic dipolar solvents with high boiling points, or product isolation through liquid-liquid extraction. In contrast to the classical method microwave irradiation often leads to a remarkable decrease in the reaction times and increased yields (in our case up to 98%). The products could be purified simply by recrystallization from an appropriate solvent or mixture of solvents. The synthesized coumarin derivatives were tested as antimicrobial agents by the disc-diffusion and MIC methods on some Gram positive and Gram negative bacteria and the fungus *C. albicans.*

## 2. Results and Discussion

### 2.1. Synthesis

Preparations of coumarin derivatives **2b**-**8b** by the conventional condensation method were characterized by lower yields of the desired compounds (no more than 32%), followed by time consuming purifications by column chromatography. Thus, we report herein substantial improvements to the synthesis of these compounds using a solvent free microwave promoted reaction. The obtained results indicated significant yield increases (up to 87%), decreases in reaction times and, as far as purification of products is concerned, requiring only recrystallization from appropriate solvents.

Also, in a case of synthesis of imino derivatives of 4-hydroxychromene-2-one (**1**) microwave heating provided several advantages compared to the conventional method of preparation by azeotropic water removal. Due to homogenous heating achievable under microwaves side reactions were almost eliminated, which resulted in increased yields of the desired compounds **9b**-**16b** (up to 90%). Additionally purifications were limited to only recrystallization from methanol, without requiring time and solvent consuming column chromatography.

Both elemental and spectral (IR, ^1^H-NMR, mass spectra) analysis data of all compounds were in full agreement with the suggested molecular structures. The IR spectra of pure products **2b**-**8b** indicated the presence of a broadened OH band in the 3437-3422 cm^-1^ region, corresponding to the hydroxyl fragment of the coumarin moiety. A strong coumarin moiety lactone C=O band at 1706-1699 cm^-1^ was also observed. Synthesized compounds **2b**, **3b**, **4b** and **5b** possess additional strong C=O bands from ester COOMe or COOEt groups at 1731-1729 cm^-1^. Additional acetyl group C=O bands were observed at 1700-1686 cm^-1^ for compounds **3b**, **6b** and **8b**. The IR spectra of compounds **7b** and **8b** showed broadened OH bands at 3111 and 3100 cm^-1^ and C=O bands at 1699-1689 cm^-1^ from COOH groups. Strong absorptions in the 2231-2229 cm^-1^ region indicated the presence of C≡N groups in the prepared compounds **5b** and **7b**. The ^1^H-NMR spectra of compounds **2b**-**8b** showed the characteristic coumarin aromatic proton signals in the 7.83-7.29 ppm range (m, 4H, C-5-H, C-6-H, C-7-H and C-8-H). All these spectra also contain resonances from methyl fragments (C=C-CH_3_, singlet at 2.26-2.21 ppm) and hydroxyl groups (OH-4, broad singlet at 17.17-16.73 ppm). The protons of the methyl group from the COOMe fragment of compounds **3b**, **4b** and **5b** were observed as singlets at 3.86-3.81 ppm, while the compound **2b** showed ethyl proton resonances at 4.21 ppm (q, 4H, CH_2_) and 1.23 ppm (m, 6H, CH_3_). In the vinyl proton region compound **4b** showed an absorption at 5.76 ppm (s, C=C-H). Since the compounds **7b** and **8b** were prepared from acetic acid derivatives, broadened OH proton peaks were observed at low field (12.37 and 12.65 ppm, respectively). The methyl protons from the acetyl fragment of compounds **3b**, **6b** and **8b** were identified in the range of 2.29-2.26 ppm.

Formation of imine derivatives **9b**-**15b** was evidenced by appearance of ^1^H-NMR signals in the aromatic protons region at 8.39-6.9 ppm and a strong C=N group IR band at 1610-1606 cm^-1^. Also, all synthesized compounds showed CH_3_-C=N proton resonances in the range of 2.81-2.61 pmm (s, CH_3_, 3H). In the ^1^H-NMR spectrum of compound **16b** a multiplet at 1.82 ppm for a –CH­_2_-CH_2_- group, two triplets at 2.46 ppm and 3.60 ppm for a methylene protons from C’-4 and C’-1, respectively, and a broadened singlet from the hydroxyl group of COOH fragment at 11.3 ppm were observed.

In Table 1 reaction conditions, substituents and yields of obtained compounds are presented. In Table 2 and Table 3 molecular descriptors and side chain dihedral angles of the compounds that had been obtained by Mopac, CAChe and Spartan [28,29,30] software, are presented.

**Figure 1:**
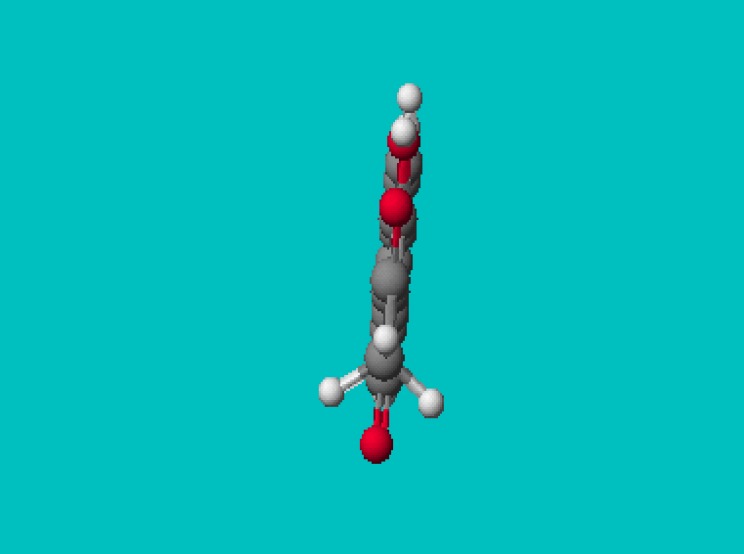
PM5 geometry of *3-acetyl-4-hydroxy-chromene-2-one* (**1**).

**Figure 2:**
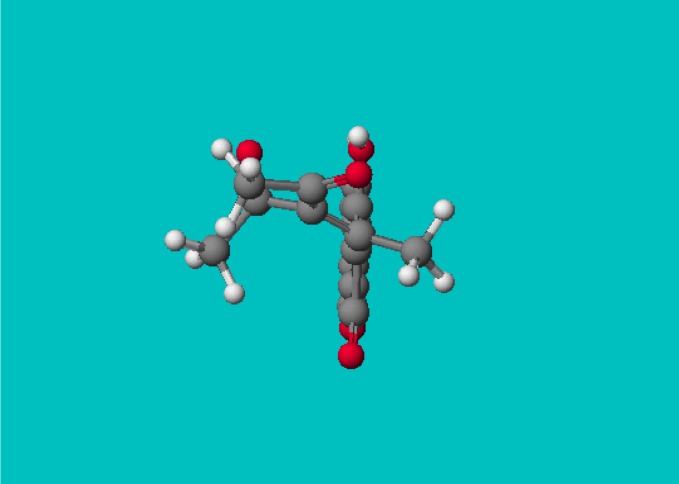
PM5 geometry of *3-(1-(4-hydroxy-2-oxo-2H-chromen-3-yl)-ethylidene)-pentane-2, 4-dione* (**6b**).

**Figure 3:**
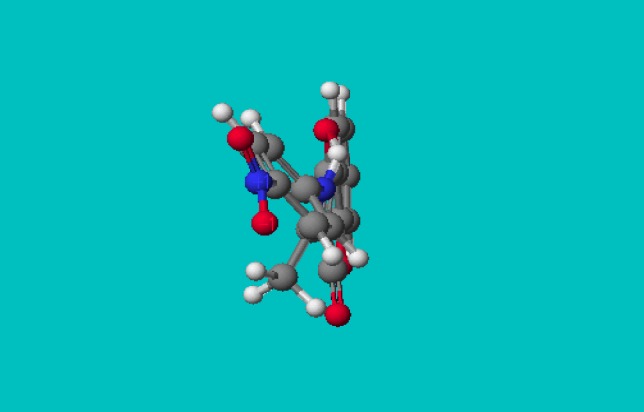
PM5 geometry of *4-hydroxy-3-(1-(4-nitrophenylimino)ethyl)-2H-chromen-2-one* (**13b**).

**Figure 4:**
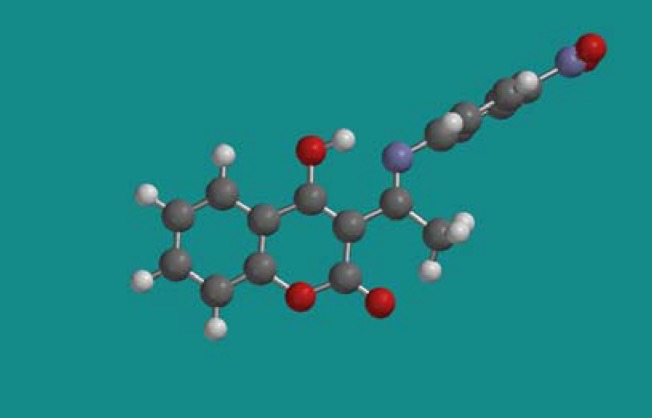
Spartan geometry of *4-hydroxy-3-(1-(4-nitrophenylimino)ethyl)-2H-chromen-2-one* (**13b**).

Compound **1**, as a base for synthetic research, with compounds **6b** and **13b**, were subjected to geometry examination since molecule planarity and voluminous side chain groups play important roles in the bioactivity of the compounds [31,32]. Geometries are presented in Figure 1, Figure 2, Figure 3 and Figure 4. The presented compounds were selected based on results that confirm influence of planarity and substituent nature when coumarins penetrate bacterial cells [31,32].

**Table 1:** Reaction conditions, substituents and yields of obtained compounds **2b**-**8b**.

No	R­_1_	R_2_	Pw (W)	Tm(S)	T^a^ (ºC)	Yield (%)	
						A	B
**2b**	COOEt	COOEt	500	7	123	27	96
**3b**	COMe	COOMe	500	7	129	36	97
**4b**	H	COOMe	500	9	121	18	96
**5b**	C≡N	COOMe	500	7	121	33	94
**6b**	COMe	COMe	500	10	134	41	94
**7b**	C≡N	COOH	500	6	120	42	84
**8b**	COMe	COOH	500	5	126	35	87
**No**	**R­_3_**		**Pw (W)**	**Tm(min)**	**T (ºC)**	**Yield (%)**	
						** A **	** B **
**9b**	Ph		500	3	109	75	95
**10b**	p-tolyl		500	3	109	73	97
**11b**	m-tolyl		500	3	109	84	94
**12b**	o-tolyl		500	3	109	73	94
**13b**	p-NO_2_-phenyl		500	3	109	51	92
**14b**	m-NO_2_- phenyl		500	3	109	62	97
**15b**	benzyl		500	3	109	75	97
**16b**	C_4_H_9_COOH		500	3	109	42	87

**Table 2:** Molecular descriptors of observed compounds.

Parameters	Method	Compounds						
		1	3b	4b	6b	7b	8b	13b
Heat of Formation (kcal/mol)	PM3	-120.57	-184.38	-149.91	-140.02	-119.39	-193.17	-20.97
	PM5	-135.31	-207.47	-166.62	-168.25	-139.00	-214.49	-64.73
	SPARTAN(e.u)	26.97	57.58	57.26	70.91	40.23	41.66	72.38
Electronic Energy (eV)	PM3	-14412.18	-26955,66	-21039,91	-25237,76	-22271,14	-25448,75	-27320,32
Core-core Repulsion (EV)	PM3	11811.55	23083.21	17482.15	21395.99	18547.35	21441.45	23332.61
Gradient Norm	PM3	414.37	430.48	447.56	383.06	115.66	69.75	320.41
Dipole (debye)	PM3	7.32	4.99	7.25	6.11	6.54	4.96	9.94
	SPARTAN	3.89	3.85	5.10	5.76	5.73	2.79	8.24
Symetry	PM3	C1	C1	C1	C1	C1	C1	C1
	SPATRAN	C1	C1	C1	C1	C1	C1	C1
No. of Fields Level	PM3	38	57	49	54	50	54	60
Ionization Potencial (eV)	PM3	9.70	9.41	9.34	9.34	9.67	9.50	9.59
Homo Lumo Energies (eV)	PM3	-9.70	-9.41	-9.34	-9.34	-9.67	-9.50	-9.60
		-1.26	-1.30	-1.37	-1.30	-1.59	-1.31	-1.45
	SPARTAN	-9.39	-9.59	-9.54	-9.66	-9.75	-9.57	-9.81
		1.26	-1.29	-1.26	-1.26	-1.50	-1.32	-1.55
Molecular Weight	PM3	204.18	302.28	260.25	286.28	271.23	288.26	342.29
Scf Calculations	PM3	10	14	102	84	71	202	10

**Table 3:** Side chain atom angles of observed compounds.

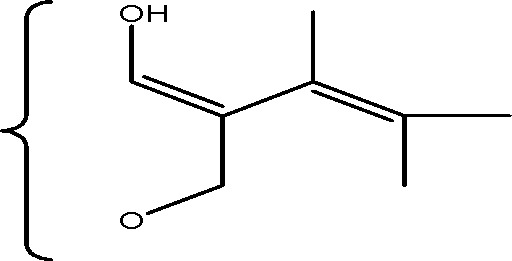	Side chain atoms	Angle (º)	Dihedral atoms	Dihedral (º)
1	-C=C-CO	120,67	C (enol from ring)-C=C-CO	0,21
3b	-C=C-CO	122,43	-C=C-C-O	-61,94
	-C=C-COO	30,95	-C=C-C-O (carbonyl)	-3,69
			-C=C-C-C (ester)	-5,48
4b	-C=-C(from ring)-C(Me)	115,93	C=C(ring)-C (side chain)-C(Me)	-127,19
	-C=C-CO	126,44	-C=C-C-O (carbonyl)	-134,44
			-C=C-C-C(ester)	47,62
6b	C=C-C(carbonyl)	121,79122,84	C=C-C-O	88,8615,34
7b	C=C-CN	123,06	C=C-C-C (cyano)	3,28
	C=C-C(carboxyl acid)	122,92	-C=C-C-O (carbonyl)	-119,99
			-C=C-C-O (ester)	61,05
8b	-C=C-CO	119,79	-C=C-C-O (carbonyl)	-54,78
	C=C-C(carboxyl acid)	124,68	-C=C-C-O (carbonyl)	154,01
			-C=C-C-O(ester)	-30,51
13b	=C(from ring)-C=N-	122,79	=C (from ring)-C=N-C (aromatic)	180

### 2.2. Minimal Inhibitory Concentration

All the reported compounds exhibited moderate *in vitro* activity against the tested bacterial strains (Table 4 and Table 5) and the yeast *C. albicans.* Solutions of compound **1** for testing against *S. aureus* (isolate) were prepared in following concentrations: 0.94, 1.90, 3.80, 7.50, 15.00 and 30.00 mg/mL. For other cultures tested the concentrations of compound **1** were 0.09, 0.19, 0.38, 0.75, 1.50 and 3.00 mg/mL. For the rest of the compounds, the concentrations were: 0.125, 0.25, 0.50, 1.00, 2.00 and 4.00 mg/mL. Tests were run in triplicate. Standards were tetracycline and ketoconazole (both at 0.500 µg/mL).

MIC values for all tested microorganism were in the range from 0.185 mg/mL to 2 mg/mL, andresults were read after 24 h and 48 h respectively. After 24 h, with MICs of 0.09 (compound **1**) and 0.13 mg/mL (compounds **3b-13b**), no bacterial growth was visible in test tubes containing *S. aureus*, indicates that all seven compounds are good growth inhibitors of this species. Some compounds were less effective on the same culture isolated from tissue and towards *M. lysodiekticus*, and they stopped the growth at a concentration of 0.50 mg/mL. After 48 hours, none of applied compounds stopped growth of *S. aureus* (isolate) or *M. lysodiekticus*, and a change of the indicator’s color was visible. Tested bacteria were most sensitive to the compound **1**, one with no substituent, and **6b**, which contains two acetyl groups. Carboxyl, ester and cyano derivatives demonstrated slightly lower activity.

**Table 4:** MIC values (mg/mL) of the synthesized coumarin derivatives.

	Cultures									
	*S. aureus*		*S. aureus*		*M. lysodiekticus*		*E. coli*		*C. albicans*	
			(isolate)							
Comp.	24 h	48 h	24 h	48 h	24 h	48 h	24 h	48 h	24 h	48 h
**1**	0.09	0.19	0.94	1.90	0.19	0.38	0.19	0.38	0.09	0.09
**3b**	0.13	0.50	0.50	1.00	0.13	0.50	0.13	0.50	0.13	0.50
**4b**	0.13	0.50	0.50	1.00	0.13	0.50	0.13	0.50	0.25	0.50
**6b**	0.13	0.25	0.13	0.50	0.50	0.50	0.25	1.00	0.13	0.25
**7b**	0.13	1.00	0.50	0.50	0.13	0.50	0.50	1.00	0.50	1.00
**8b**	0.13	0.50	0.50	1.00	0.13	0.50	0.13	0.50	0.13	0.13
**13b**	0.13	0.50	0.13	0.50	0.50	0.50	0.13	0.50	0.13	0.13

The lowest sensitivity, only for *S. aureus*, was displayed by the derivative **7b** with a cyano group (MIC 1 mg/mL). Different compounds permitted growth of *M. lysodeikticus* and *S. aureus* (isolate), with MIC values ranging from 0.38 to 0.50 mg/mL. Schiff-base’ inhibitory potential was very similar to compounds **3b**, **4b** and **8b**, for all of bacteria, regardless of differences in structure. It is interesting, regarding the *S. aureus* results after 48 h, that with an increase of the HOMO values of the synthesized compounds from most negative (- 9.67) to less (- 9.37), the MIC values decreased. It was concluded that coumarin derivatives acting as electrophilic agents have a positive effect and inhibited bacterial growth [35,36]. Similar MICs were obtained for *M. lysodiekticus*. A LUMO decrease resulted in a MIC increase, with the exception of compound **6b**, as result of the better coumarin derivative penetration in bacterial cells in the absence of coumarin molecule nucleophilic action [35,36].

*E. coli* was also tested as an example of Gram negative bacteria, and only compounds **6b** and **7b** permitted growth after 24 h. For the rest of the applied compounds the MIC was 0.13 mg/mL. Results showed that after 48 h the best drug for Gram positive bacteria (**6b**) was the worst one for Gram negative. After 48 h, the minimum inhibitory concentration of compound **6b** was two to four times higher within Gram negative than Gram positive bacteria. Also, the earlier mentioned relationship between HOMO, LUMO and MIC was the opposite for *E. coli*, but that was expected due to differences in the chemical constitution of Gram positive and negative bacteria cell walls and the different mechanisms of coumarin penetration in bacteria cells [35,36].

Analysis further showed that only compounds **4b** and **7b** allowed growth of *C. albicans* after 24 h. While compounds **1**, **8b** and **13b** still prevented the growth of fungus at a concentration of 0.13 mg/mL, after 48 hours, the rest of the tested drugs showed a lack of inhibitory action, which is very interesting due to similarity of their structure. That was conformation that, in general, derivatives with carboxyl, ester or cyano group, presented slightly lower drug potential than acetyl-substituded ones.

Finally, the *p*-nitrophenylimino derivative of coumarin is a completely different pharmacophore, containing with two planar systems (Figure 4), and p-nitro group attached on imino ring. For all tested cultures of bacteria the final MIC was the same: 0.5 mg/mL. That indicates that mechanism of interaction with bacteria is very similar. Also, compound **13b** is very powerful growth inhibitor of fungus.

### 2.3. Cylinder plate diffusion method

The results of diffusion antimicrobial activity results are presented in Table 5. 

**Table 5:** Cylinder plate diffusion method (µg/mL) of synthesized derivatives.

		Cultures ^a^													
I		II		III		IV		V		VI		VII	
Time (h)		24	48	24	48	24	48	24	48	24	48	24	48	24	48
Comp.	Conc.(µg/mL)	Zones of inhibition (mm) ^b,c,d^													
**1**	75	15	16	10	10	8	8	6	7	17	17	10	12	26	30
**10b**	75	/	/	/	/	/	/	/	/	/	/	/	/	/	/
	150	/	/	/	/	/	/	/	/	/	/	/	/	/	/
**13b**	75	/	/	/	/	/	/	/	/	/	/	/	/	22	23
	150	/	/	/	/	/	/	/	/	/	/	/	/	30	37
**16b**	75	12	13	15	16	/	/	7	8	16	16	8	10	20	25
**Antibiotic**	10	26	24	20	19	24	25	52	53	24	23	21	21	26	23

^a^ I *S. aureus*; II *S. aureus* (isolate); III *M. lysodiecticus*,;IV *K. pneumonia*; V *B. subtilis*, VI *E. coli*; VII *C. albincans*; ^b^ (/) means absence of an inhibition zone; the results are from experiments run in triplicate, SD=±3 mm; ^c^ ethanol solvent controls were negative; ^d^ Standards were tetracycline (10 mg/mL) and ketoconazole (10 mg/mL).

Disc diffusion values for all tested microorganism were in the range 75-150 µg/mL. Only compound **1** and selected Schiff bases have been tested as possible drugs by this method. The main result of the experiment is that compound **13b** at a concentration of 150 µg/mL presented better inhibitory potential than applied antibiotic for *C. albicans* (30-37 mm). This molecule, with two planar systems [31,32,33,34] and a *p*-nitro group (Figure 5) has a great effect on fungus cell wall [37] and, therefore, totally lack of fungus resistance. It is very interesting that compound **10b** with very similar structure but without a *p*-nitro group, showed complete lack of inhibitory activity for all tested cultures.

According to our test results, only compound **1** demonstrated inhibitory potential for all tested cultures within 24 hours of testing. When testing was performed on *C. albicans*, the inhibition zone of compound **1** was similar to that of **13b**. All examined microorganisms were sensitive to the presence of compound **16b**, except *M. lysodiecticus*. Measured zones of inhibition, both for Gram positive and Gram negative bacteria, showed that compounds **1** and **16b** are less effective than applied antibiotics. These compounds have no subtitution or an *n*-pentanoic acid moiety as substituent.

The tested cultures of bacteria showed notable resistance to the other compounds at the applied concentrations. It appears that only those compounds with free carbonyl or carboxyl groups present some growth inhibitory potential in 6-20 mm range within 24 and 48 hours. In general, the less sensitive culture was *K. pneumoniae* (Gram negative); and the most sensitive one was *B. subtilis* (Gram positive). Results for the fungus were very different. The behavior of compounds **1** and **16b** was similar and they both stopped the growth of *C. albicans*, but the measured inhibition zones were larger than with tested bacteria, and conclusion is that the fungus was more sensitive to these compounds.

The general antimicrobial activity of natural coumarins has been documented in woodruff (*Galium odoratum*) extract [33]; the MIC values of some natural coumarins against Gram positive bacteria were 62.5 g/mL-125 g/mL, suggesting that phenyl chain of position 8 and hydroxyl group at position 7 of the benzene ring are required for activity against *E. coli* and *S. aureus*. The antifungal potential of coumarins can be related to the presence of an alkyl group at the C-8 position and the MIC for these compounds for *C. albicans* was about 250 g/mL [34]. Planarity of the coumarin ring makes penetration in Gram positive bacteria easier, but other factors such as shape have to be considered as well [31,32,33,34]. Coumarins proved to be slightly less active against Gram negative bacteria, due possibility to particular physical properties [31] (and in agreement with the results seen here).

## 3. Experimental

### 3.1. Chemistry

**Scheme 1:**
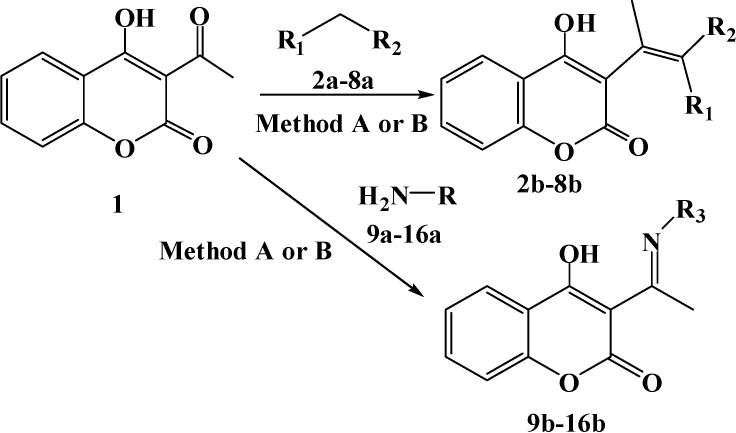
Reaction of Condensation of 3-acetyl-4-hydroxy-chromene-2-one (**1**) with carbonyl compounds **2a**-**8a** and amines **9a**-**16a** (A. Conventional method; B. Microwave method).

The sequence of the reactions used for the synthesis of the novel 4-hydroxychromene-2-one derivatives is outlined in Scheme 1. Compounds **2b-8b** had been synthesized by Knoevenagel condensation using both conventional and fast microwave procedures. Different carbonyl, ester and cyano derivatives were used in the condensations with 4-hydroxychromene-2-one to achieve structural variety in the produced coumarins. Synthesis of compounds **9b-15b** presents easy preparation of coumarin Schiff-base derivatives, also by two methods of preparation. The goal of synthesis was preparation of both aromatic and aliphatic 4-hydroxy-chromene-2-one derivatives and theirs characterization as potential antimicrobial agents. Both methods are simple with short reaction times and high yields.

### 3.2. Molecular Descriptors

Geometries of the compounds were refined by three different semi-empirical methods: PM3 from Mopac 2000 [28], version 11 (Chem 3D v9, 2006) with gradient norm 0.100; PM5 method from CAChe WorkSystem Pro 6.01 software, and Minimize Energy method from Spartan 2002 for Windows. List of the descriptors is presented in Table 2. Also, in Table 3 we present side chain atom angles and dihedral angles for the compounds, calculated and optimized with Spartan 2002 tools. Figures with PM5 geometries of selected compounds were taken by CAChe software.

### 3.3. Microbiology

#### 3.3.1. Culture of Microorganisms

Test bacteria used in this experiment were: *Staphylococcus aureus* (ATCC 25925), *Staphylococcus aureus* (clinical isolate, IHP), *Escherichia coli* (ATCC 25922), *Micrococcus lysodeikticus* (ATCC 4698) and *Candida albicans* (ATCC 10259), *Bacillus subtilis* (clinical isolate, IHP), *Klebsiela pneumoniae* (clinical isolate, IHP). All tested bacteria and fungi *C. albicans* were obtained from Laboratory for Microbiology, Faculty of Chemistry, University of Beograd, Serbia, and the Laboratory for Microbiology, from Institute for Health Protection (IHP) in Kragujevac.

#### 3.3.2. Minimal Inhibitory Concentration

The antimicrobial activity of synthesized coumarins was measured using the microdilution method in Mueller-Hinton broth [38,39]. The microdilution method was performed using a 24 h old culture of bacteria (and 48 h old culture of *C. albicans*) which was reseeded on the nutrient broth at a temperature of 37 °C. The inoculums were made and concentrations were adjusted with sterile water to 5.6 x 10^6^ CFU/mL for bacteria and 3 x 10 ^4 ^ CFU/mL for fungus. A series of two-fold dilutions of the coumarins, ranging from 4 mg/mL to 0.125 mg/mL, was prepared in Mueller-Hinton broth with the addition of 0.1 mL of bacterial (yeast) suspensions. A set of tubes containing only inoculated broth was kept as a control. Tetracycline and ketoconazole were used as the control drugs. MICs were determined as the lowest concentration of the compounds that inhibited visible growth, and by the changing color of Resazurin solution (0.02 mL of 0.05% concentration) from blue to pink [40].

#### 3.3.3. Disc diffusion Cylinder Plate Method

Muller Hinton Agar (for bacteria) and Sabouraud dextrose agar (for yeast) were prepared with the addition of 1 mL of microbial suspension (6.5 x 10^6^ CFU/mL for bacteria and 3 x 10^4^ CFU/mL for yeast) and the plates incubated at 37^o^ C for approximately 20 min. Then sterile vertical cylinders were placed on the Petri dishes and samples of compounds (75 µg/mL and 150 µg/mL in ethanol) were aseptically poured into the vertical cylinder using micropipettes [41,42,43,44]. The plates were subsequently incubated for 24 hours at 37^º^C for bacteria and 48 hours at 28^º^C for yeast. The diameter of zones of inhibition was measured in mm. The standard antibiotics were tetracycline for bacteria and ketoconazole for *C. albicans.*

### 3.4. Instruments and apparatus

All reagents, solvents and catalyst were of analytical grade and used directly. All the melting points were recorded on a Kofler-hot stage apparatus (C. Reichert, Vienna, Austria) and uncorrected. The purity of compounds was checked routinely by TLC using a Merck Kieselgel 60 PF_254+366_ and a Merck Kieselgel G. and spots were visualized by exposing the dry plates in iodine vapors. Also, the purity was checked by gas-chromatography/mass spectrometry. The IR spectra were run on a Perkin-Elmer Grating Spectrophotometers Model 137 and Model 337 in cm^– 1^ (Perkin Elmer, Beaconsfield, UK). The NMR spectra were recorded on a Varian Gemini 200 spectrometer (^1^H at 200 MHz; Varian Inc., Palo Alto, CA, USA), in CDCl_3_ as solvent, using TMS (SiMe_4_) as the internal standard. Chemical shifts are given in ppm, abbreviations: s-singlet, d-doublet, t-triplet, q-quartet, ABq-AB quartet, m-multiplet. Microanalysis of carbon, hydrogen, and nitrogen was carried out with a Carlo Erba 1106 microanalyser (Carlo Erba, Milan, Italy). GC/MS were carried out in Agilent 6890N/5975B gas chromatograph. The microwave assisted reactions were carried out in a MICROSYNTH Microwave Synthesis System (serial number: 130486, terminal: T640, serial number: 0804000100), manufactured by Milestone Inc. 25 Controls Dr. Shelton, CT 06484, USA [the microwaves are generated by a magnetron (serial number: 133462/13346), at the frequency of 2450 MHz having an output energy range of 100-500 watts]. This apparatus is well suited for stringent reaction conditions, namely, anhydrous atmosphere, controlled temperature (using a fiber optic as an individual sensor for temperature control) and attachment of reflux condenser with constant stirring.

### 3.5. Preparation of 3-acetyl-4-hydroxy-chromene-2-one derivatives (general procedure)

#### 3.5.1. Knoevenagel condensation of 3-acetyl-4-hydroxy-chromene-2-one (**1**) with carbonyl compounds **2a-8a**

##### 3.5.1.1. Method A - Conventional procedure

A solution of 3-acetyl-4-hydroxychromene-2-one (**1**, 2.5 mmol) and toluene (50 mL) containing a catalytic amount of ammonium acetate was taken and carbonyl compound **2a-8a** was added. The reaction mixture was stirred for a period of 8 h at the boiling point of the solvent with azeotropic removal of water formed. The excess solvent was distilled off and solid product was filtered, dried, purified via column chromatography (benzene-acetone=8:2) to give products **2b-8b**.

##### 3.5.1.2. Method B - Microwave method

A well ground mixture of a 3-acetyl-4-hydroxychromene-2-one (**1**, 2.5 mmol), carbonyl compound **2a-8a** (2.8 mmol) and ammonium acetate (catalytic amount) was irradiated and heated in the microwave reactor at the power setting and for the time indicated in Table 1. At the end of exposure to microwaves, the reaction mixture was cooled to room temperature, and the crude product was recrystallized from methanol to afford the coumarin derivatives **2b**-**8b** (Scheme 1, Table 1).

#### 3.5.2. Condensation of 3-acetyl-4-hydroxy-chromene-2-one (**1**) with amines **9a**-**16a**

##### 3.5.2.1. Method A - Conventional method

A mixture of 3-acetyl-4-hydroxychromene-2-one (**1**, 0.01 mol), amine **9a-16a** (0.01 mol) and a catalytic amount of *p*-toluenesulfonic acid in toluene (50 mL) was heated with azeotropic removal of water for a period of 10-12 h. Progres od reaction was monitored by TLC (toluene-acetone = 7:3). At the end of the reaction, the solvent was removed under reduced pressure. The solid products were filtered, dried and recrystallized from methanol to give compounds **9b**-**16b**.

##### 3.5.2.2. Method B - Microwave method

A catalytic amount of *p*-toluenesulfonic acid was added to a toluene solution (50 mL) of equimolar amounts (0.01 mol) of 3-acetyl-4-hydroxychromene-2-one (**1**) and amine **9a-16a**. The mixture was heated under microwaves for 3 minutes. After cooling, the solvent was removed, and the obtained solid was filtered and recrystallized from methanol (Table 1, Scheme 1).

### 3.6. Spectral data of synthesized coumarin derivatives

*Diethyl 2-(1-(4-hydroxy-2-oxo-2H-chromen-3-yl)ethylidene)malonate* (**2b**): Yield: 96%; m..p. 224-226 ºC; IR (KBr, cm^-1^): ν_(OH)_ 3434, ν_(CH3 and CH2)_ 2949 and 2841, ν_(C=O)_1731 (lactone and ester), ν_(C=C)ar_, 1611, 1546 and 1496, ν_(C-O-C)_ 1368 and 1032; ^1^H-NMR (δ ppm): 1.23 (m, 6H, CH_3_, mixture of *Z* and *E* isomers), 4.21 (q, 4H, 4CH_2_), 7.31-7.83 (m, 4H, H-5, H-6, H-7, H-8), 16.73 (bs, 1H, OH-4); MS: m/z (%): 346 (M^+^); Anal. for C_18_H_18_O_7_ (346.33) (%): C: 62.42, H: 5.24; found C: 62.44, H: 5.23.

*Methyl 2-acetyl-3-(4-hydroxy-2-oxo-2H-chromen-3-yl)but-2-enoate* (**3b**): Yield: 97%; m.p. 237-239 ºC; IR (KBr, cm^-1^): ν_OH_ 3433, ν_(CH3)_, 2948, 2930, 2856, ν_(C=O)_ 1730 (lactone and ester), ν_(C=O)_ 1700, ν_(C=C)ar_ 1610, 1545, 1495 1369, ν_(C-O-C)_ 1032 and 1024; ^1^H-NMR (δ ppm): 2.20 (s, 3H, CH_3_), 2.26 (s, 3H, CO-CH_3_), 3.81 (s, 3H, COO-CH_3_), 7.29-7.83 (m, 4H, H-5, H-6, H-7, H-8), 17.01 (bs, 1H, OH-4); MS: m/z (%): 302 (M^+^); Anal. for C_16_H_14_O_6_ (302.28) (%): C: 63.57, H: 4.67; found C: 63.55, H: 4.71.

*Methyl 3-(4-hydroxy-2-oxo-2H-chromen-3-yl)but-2-enoate* (**4b**): Yield: 96%; m.p. 202-203 ºC, m.p. 222-223 ºC; IR (KBr, cm^-1^): ν_(OH)_ 3422, ν_(CH3)_ 2949, 2930, ν_(C=O)_ 2841, 1729 (lactone and ester), ν_(C=C)ar_ 1609, 1542, 1491, ν_(C-O-C)_ 1364, 1033, 1023; ^1^H-NMR (δ ppm): 2.22 (s, 3H, CH_3_), 3.86 (s, 3H, COO-CH_3_), 5.76 (s, 1H, C=C-H), 7.29-7.82 (m, 4H, H-5, H-6, H-7, H-8), 17.11 (bs, 1H, OH-4); MS: m/z (%): 260 (M^+^); Anal. for C_14_H_12_O_5_ (260.24) (%): C: 64.61, H: 4.65; found C: 64.63, H: 4.69.

*Methyl 2-cyano-3-(4-hydroxy-2-oxo-2H-chromen-3-yl)but-2-enoate* (**5b**): Yield: 94%; m.p. 247-249 ºC; IR (KBr, cm^-1^): ν_(OH)_ 3434, ν_(CH3)_ 2949, 2929, 2854, ν_(C≡N)_ 2231, ν_(C=O)_ 1731 (lactone and ester), ν_(C=C)ar_ 1611, 1546, 1497, ν_(C-O-C)_ 1368, 1032, 1025; ^1^H-NMR (δ ppm): 2.23 (s, 3H, CH_3_), 3.80 (s, 3H, COO-CH_3_), 7.29-7.83 (m, 4H, H-5, H-6, H-7, H-8), 17.06 (bs, 1H, OH-4); MS: m/z (%): 285 (M^+^); Anal. for C_15_H_11_NO_5_ (285.06) (%): C: 63.16, H: 3.89, N: 4.91; found C: 63.18, H: 3.84, N: 4.95.

*3-(1-(4-Hydroxy-2-oxo-2H-chromen-3-yl)-ethylidene)-pentane-2,4-dione* (**6b**): Yield: 94%; m.p. 241-243 ºC; IR (KBr, cm^-1^): ν_(OH)_ 3434, ν_(CH3)_ 2949, 2929, ν_(C=O)_ 1731 (lactone), ν_(C=O)_ 1697, 1686, ν_(C=C)ar_ 1610, 1544, 1496, ν_(C-O-C)_ 1371, 1032; ^1^H-NMR (δ ppm): 2.23 (s, 3H, CH_3_), 2.29 (s, 3H, CO-CH_3_), 7.31-7.83 (m, 4H, H-5, H-6, H-7, H-8), 17.01 (bs, 1H, OH-4); MS: m/z (%): 286 (M^+^); Anal. for C_16_H_14_O_5_ (286.28) (%): C: 67.13, H: 4.93; found C: 67.15, H: 4.87.

*2-Cyano-3-(4-hydroxy-2-oxo-2H-chromen-3-yl)but-2-enoic acid* (**7b**): Yield: 84%; m.p. 256-257 ºC; IR (KBr, cm^-1^): ν_(OH)_ 3433 (coumarin), ν_(OH)_ 3100 (COOH), ν_(CH3)_ 2947, 2927, ν_(C=O)_ 1731, ν_(C=O)_ 1699 (COOH), ν(C=C)_ar_ 1611, 1543’ 1495, ν_(C-O-C)_ 1371, 1032; ^1^H NMR (δ ppm): 2.24 (s, 3H, CH_3_), 7.31-7.83 (m, 4H, H-5, H-6, H-7, H-8), 12.37 (bs, 1H, COOH), 17.13 (bs, 1H, OH-4); MS: m/z (%): 271 (M^+^); Anal. for C_14_H_9_NO_5_ (271.22) (%): C: 62.00, H: 3.34, N: 5.16; found C: 62.05, H: 5.11, N: 5.21.

*2-Acetyl-3-(4-hydroxy-2-oxo-2H-chromen-3-yl)but-2-enoic acid* (**8b**): Yield: 87%; m.p. 229-231 ºC; IR (KBr, cm^-1^ ): ν_(OH)_ 3437 (coumarin), ν_(OH)_ 3111 (COOH), ν_(CH3)_ 2991, ν_(C=O)_ 1727 (lactone), ν_(C=O)_ 1699, 1689 (COOH and keto group), ν(C=C)_ar_ 1601, 1541, 1491, ν_(C-O-C)_ 1369, 1031; ^1^H-NMR (δ ppm): 2.21 (s, 3H, CH_3_), 2.28 (s, 3H, CO-CH_3_), 7.31-7.83 (m, 4H, H-5, H-6, H-7, H-8), 12.65 (bs, 1H, COOH), 17.17 (bs, 1H, OH-4); MS: m/z (%): 288 (M^+^); Anal. for C_15_H_12_O_6_ (271.22) (%): C: 62.50, H: 4.20; found C: 62.55, H: 4.21.

*4-Hydroxy-3-(1-(phenylimino)ethyl)-2H-chromen-2-one* (**9b**): Yield: 95%; m.p. 169-171 ºC; IR (KBr, cm^-1^): ν_(OH)_ 3415, ν_(=CHar,)_ 3073, 3037, ν_(CH3)_ 2929, 2853, ν_(C=O)_ 1704 (lactone), ν_(C=N)_ 1609 (imino), ν_(C=C)ar_ 1592, 1561, 1480; ^1^H-NMR (δ ppm): 2.72 (s, 3H, CH_3_-C=N), 7.3 (m, 1H, C-6-H), 7.4 (dd, 1H, C-8-H, ^3^J_8,7_=8.3 Hz, ^4^J_8,6_=1.1 Hz), 7.6 (dd, 1H, C-5-H, ^3^J_5,6_=7.8 Hz, ^4^J_5,7_=1.7 Hz), 7.7 (m, 1H, C-7-H), 6.9-7.21 (m, 5H, phenyl), 16.15 (bs, 1H, OH-C-4); MS: m/z (%): 279 (M^+^, 64), 278 (100), 264 (17), 262 (10), 250 (5), 236 (2), 234 (3), 222 (2), 188 (19), 187 (28), 158 (12), 144 (4), 131 (9), 130 (14), 121 (19), 118 (10), 93 (12), 92 (6), 77 (25), 65 (6), 51 (8); Anal. for C_17_H_13_NO_3_ (279.29) (%): C: 73.11, H: 4.69, N: 5.02; found C: 73.09, H: 4.71, N: 4.97.

*4-Hydroxy-3-(1-(p-tolylimino)ethyl)-2H-chromen-2-one* (**10b**): Yield: 97%; m.p. 147-149 ºC. IR (KBr, cm^-1^): ν_(OH)_ 3421, ν_(=CHar,)_ 3073, ν_(CH3)_ 2985, 2922, 2852, ν_(C=O)_ 1709 (lactone), ν_(C=N)_ 1611 (imino), ν_(C=C)ar_ 1597, 1569, 1513, 1483; ^1^H-NMR (δ ppm): 2.41 (s, 3H, CH_3_-C’-3), 2.69 (s, 3H, CH_3_-C=N), 7.09-7.65 (AB_q_, 4H, phenyl, ^3^J=8.43 Hz 7.3 (m, 1H, C-6-H), 7.4 (dd, 1H, C-8-H, J_8,7_=8.3 Hz, J_8,6_=1.1 Hz), 7.6 (dd, 1H, C-5-H, J_5,6_=7.8 Hz, J_5,7_=1.7 Hz), 7.7 (m, 1H, C-7-H), 16.07 (bs, 1H, OH-C-4); MS: m/z (%): 293 (M^+^, 91), 292 (100), 278 (32), 276 (20), 264 (5), 236 (2), 188 (21), 187 (12), 172 (9), 158 (5), 145 (5), 144 (12), 132 (10), 121 (23), 107 (12), 106 (11), 91 (23), 77 (8), 65 (16), 51 (3); Anal. for C_18_H_15_NO_3_ (293.32) (%): C: 73.71, H: 5.15, N: 4.78: found C: 73.72, H: 5.12, N: 4.79.

*4-Hydroxy-3-(1-(m-tolylimino)ethyl)-2H-chromen-2-one* (**11b**): Yield 94%; m.p. 109-110 ºC. IR (KBr, cm^-1^): ν_(OH)_ 3417, ν_(=CH)ar_ 3067, ν_(CH3)_ 2982, 2929, 2853, ν_(C=O)_ 1697 (lactone), ν_(C=N)_ 1606 (imino), ν_(C=C)ar_ 1600, 1566, 1484; ^1^H-NMR (δ ppm): 2.42 (s, 3H, CH_3_-C’-3), 2.70 (s, 3H, CH_3_-C=N), 7.01 (dd, 1H, C-4’-H, ^3^J_4’,5’_=7.58 Hz, ^4^J_4’,6’_=1.14 Hz,), 7.04 (dd, 1H, C-6’-H, ^3^J_6’,5’_=8.12 Hz, ^4^J_6’,4’_=1.14 Hz), 7.12 (s, 1H, C-2’-H), 7.25 (dd, 1H, ^3^J_5’,4’_=7.58 Hz, C-5’-H, ^3^J_5’,6’_=8.12 Hz), 7.3 (m, 1H, C-6-H), 7.4 (dd, 1H, C-8-H, ^3^J_8,7_=8.3 Hz, ^4^J_8,6_=1.1 Hz), 7.6 (dd, 1H, C-5-H, ^3^J_5,6_=7.8 Hz, ^4^J_5,7_=1.7 Hz), 7.7 (m, 1H, C-7-H), 15.9 (bs, 1H, OH-C-4); MS: m/z (%): 293 (M^+^, 81), 292 (100), 278 (30), 276 (12), 264 (3), 236 (3), 188 (14), 187 (27), 172 (11), 158 (7), 145 (13), 144 (12), 132 (10), 121 (19), 107 (12), 106 (6), 91 (20), 77 (9), 65 (15), 51 (4); Anal. for C_18_H_15_NO_3_ (293.32) (%): C: 73.71, H: 5.15, N: 4.78; found C: 73.72, H: 5.14, N: 4.79.

*4-Hydroxy-3-(1-(o-tolylimino)ethyl)-2H-chromen-2-one* (**12b**): Yield 94%; m.p. 138-139 ºC. IR (KBr, cm^-1^): ν_(OH)_ 3466.94, ν_(=CH)ar_ 3072.51, ν_(CH3)_ 2935.08, 2856.34, ν_(C=O)_ 1711.85 (lactone), ν_(C=N)_ 1610.82 (imino), ν_(C=C)ar_ 1594.26, 1562.86, 1486.38; ^1^H-NMR (δ ppm): 2.30 (s, 3H, CH_3_-C-1’), 2.81 (CH_3_-C=N), 7.23 (dd, 1H, C-6’-H, ^3^J_6’,5’_=8.01 Hz, ^4^J_6’,4’_=1.12 Hz), 7.28 (dd, 1H, C-3’-H, ^3^J_3’,4’_=7.62 Hz, ^4^J_3’,5’_=1.11 Hz), 7.3 (m, 1H, C-6-H), 7.4 (dd, 1H, C-8-H, ^3^J_8,7_=8.3 Hz, ^4^J_8,6_=1.1 Hz), 7.37 (m, 1H, C-5’-H), 7.6 (dd, 1H, C-5-H, ^3^J_5,6_=7.8 Hz, ^4^J_5,7_=1.7 Hz), 7.7 (m, 1H, C-7-H), 15.8 (bs, 1H, OH-C-4); MS: m/z (%): 293 (M^+^, 42), 292 (34), 278 (100), 188 (7), 187 (11), 172 (8), 158 (15), 145 (4), 144 (9), 132 (9), 121 (16), 107 (2), 106 (3), 91 (17), 77 (6), 65 (13), 51 (6); Anal. for C_18_H_15_NO_3_ (293.32) (%): C: 73.71, H: 5.15, N: 4.78: found C: 73.75, H: 5.21, N: 4.73.

*4-Hydroxy-3-(1-(4-nitrophenylimino)ethyl)-2H-chromen-2-one* (**13b**): Yield 92%; m.p. 212-215 ºC. IR (KBr, cm^-1^): ν_(OH)_ 3414, ν_(=CH)ar_ 3081, 3046, ν_(CH3)_ 2992, 2947, 2849, ν_(C=O)_ 1707 (lactone), ν_(C=N)_ 1608 (imino), ν_(C=C)ar_ 1580, 1519, 1483, ν_ar(NO2)_ 1557, 1340; ^1^H-NMR (δ ppm): 2.76 (s, 3H, CH_3_-C=N), 7.3 (m, 1H, C-6-H), 7.4 (dd, 1H, 8-H, J_8,7_=8.3 Hz, J_8,6_=1.1 Hz), 7.43 (d, 2H, ^3^J_3’, 2’_=^3^J_5’,6’_=8.9 Hz, 3’-H, C-5’-H), 7.6 (dd, 1H, C-5-H, ^3^J_5,6_=7.8 Hz, ^4^J_5,7_=1.7 Hz), 7.7 (m, 1H, C-7-H), 8.39 (d, 2H, ^3^J_2’,3’_=J_6",5"_=8.9 Hz, C-2’-H, C-6’-H), 15.95 (bs, 1H, OH-C-4); MS: m/z (%): 324 (M^+^, 60), 323 (100), 309 (8), 307 (10), 294 (14), 277 (18), 263 (8), 221 (1), 207 (7), 203 (6), 188 (26), 187 (47), 176 (6), 163 (9), 157 (9), 121 (48), 117 (12), 108 (7), 92 (14), 77 (9), 76 (13), 67 (14), 65 (12); Anal. for C_17_H_12_N_2_O_5_ (324.29) (%): C: 62.96, H: 3.73, N: 8.64: found; C: 62.97, H: 3.77, N: 8.69.

*4-Hydroxy-3-(1-(3-nitrophenylimino)ethyl)-2H-chromen-2-one* (**14b**): Yield: 97%; m.p. 209-210 ºC. IR (KBr, cm^-1^): ν_(OH)_ 3416, ν_(=CH)ar_ 3089, 3062, ν_(CH3)_ 2980, 2936, 2853, ν_(C=O)_ 1705 (lactone), ν_(C=N)_ 1609 (imino), ν_(C=C)ar_ 1590, 1538, 1491, ν_ar(NO2)_ 1562, 1353; ^1^H-NMR (δ ppm): 2.61 (s, 3H, CH_3_-C=N), 7.26 (dd, 1H, 6’-H, J_6’,5’_=8.02 Hz, ^4^J_6’,4’_=1.12 Hz), 7.3 (m, 1H, C-6-H), 7.34 (s, 1H, C-2’-H), 7.4 (dd, 1H, C-8-H, ^3^J_8,7_=8.3 Hz, ^4^J_8,6_=1.1 Hz), 7.54 (m, 1H, C-5’-H), 7.6 (dd, 1H, C-5-H, ^3^J_5,6_=7.8 Hz, ^4^J_5,7_=1.7 Hz), 7.7 (m, 1H, C-7-H), 8.09 (dd, 1H, C-4’-H, ^3^J_4’,5’_=8.5 Hz, ^4^J_4’,6’_=1.12 Hz), 16.02 (bs, 1H, OH-C-4); MS: m/z (%): 324 (M^+^ 69), 323 (83), 307 (100), 294 (14), 263 (61), 249 (3), 234 (3), 220 (3), 207 (7), 188 (26), 187 (67), 176 (6), 163 (15), 157 (12), 145 (5), 138 (5), 130 (8), 121 (78), 117 (19), 103 (9), 92 (27), 77 (15), 76 (29), 67 (25), 65 (18), 51 (6); Anal. for C_17_H_12_N_2_O_5_ (324.29) (%): C: 62.96, H: 3.73, N: 8.64: found: C: 62.96; H: 3.73; N: 8.64.

*3-(1-(Benzylimino)ethyl)-4-hydroxy-2H-chromen-2-one* (**15b**): Yield 97%; m.p. 151-152 ºC. IR (KBr, cm^-1^): ν_(OH)_ 3406, ν_(=CH)ar_ 3032, 3012, ν_(CH2)_ 2930, ν_(C=O)_ 1698 (lactone), ν_(C=N)_ 1612 (imino), ν_(C=C)ar_ 1586, 1572, 1485; ^1^H-NMR (δ ppm): 2.64 (s, 3H, CH_3_-C=N), 3.95 (t, 2H, CH_2_-N=C), 7.29 (m, 2H, C’-3-H, C’-5-H), 7.26 (m, 1H, C’-4-H), 7.39 (dd, 2H, C-2’-H, C-6’-H, ^3^J_2’,3’_=^3^J_6’,5’_=7.7 Hz, ^4^J_2’,4’_=^4^J_6’,4’_=1.1 Hz), 7.3 (m, 1H, C-6-H), 7.4 (dd, 1H, C-8-H, ^3^J_8,7_=8.3 Hz, ^4^J_8,6_=1.1 Hz), 7.6 (dd, 1H, C-5-H, ^3^J_5,6_=7.8 Hz, ^4^J_5,7_=1.7 Hz), 7.7 (m, 1H, C-7-H), 16.01 (bs, 1H, OH-C-4); MS: m/z (%): 293 (M^+^, 100), 292 (14), 276 (15), 262 (2), 236 (3), 202 (66), 189 (6), 173 (10), 156 (19), 144 (14), 131 (9), 121 (16), 107 (12), 106 (6), 91 (20), 77 (9), 65 (15), 51 (4); Anal. for C_18_H_15_NO_3_ (293.32) (%): C: 73.71, H: 5.15, N: 4.78; found C: 73.73; H: 5.13; N: 4.73.

*5-(1-(4-Hydroxy-2-oxo-2H-chromen-3-yl)ethylideneamino)pentanoic acid* (**16b**): Yield 87%; m.p. 169-171 ºC; IR (KBr, cm^-1^): ν_(OH)_ 3418 (coumarin), ν_(OH)_ 3602-2811 (COOH), ν_(CH3,CH2)_ 2947, 2930, 2875, ν_(C=O)_ 1721 (COOH), ν_(C=O)_ 1703 (lactone), ν_(C=N)_ 1614 (imino), ν_(C=C)ar_ 1600, 1560, 1487; ^1^H-NMR (δ ppm): 1.82 (m, 4H, C-2’-H, C’-3-H), 2.46 (t, 2H, C’-4-H, ^3^J_4’,3’_=7.07 Hz), 2.70 (s, 3H, CH_3_-C=N), 3.60 (t, 2H, C’-1-H, ^3^J_1’,2’_=7.1 Hz), 7.3 (m, 1H, C-6-H), 7.4 (dd, 1H, C-8-H, ^3^J_8,7_=8.3 Hz, ^4^J_8,6_=1.1 Hz), 7.6 (dd, 1H, C-5-H, ^3^J_5,6_=7.8 Hz, ^4^J_5,7_=1.7; MS: m/z (%)=303 (M^+^); Anal. for C_16_H_17_NO_5_ (303.11) (%): C: 63.36; H: 5.65; N: 4.62; found C: 63.27; H: 5.68; N: 4.63.

## 4. Conclusions

New coumarin derivatives have been synthesized using conventional and microwave heating methodology and characterized. The advantages in the use of microwave methodology are shorter reaction times, higher yields and simplified work up procedures for the point of purification of the prepared compounds. All tested compounds are in principal very good potential microorganism growth inhibitors, but their structural diversity resulted in great differences in inhibitory potential. Finally, a nice result of this experiment is that we have synthesized some compounds that have varied and different influence on different classes of bacteria and the fungus *C. albicans.*
